# Draft Genome Sequence of Salmonella enterica subsp. enterica Serovar Enteritidis MS 501, a Potential Human Pathogen Isolated from Red Lettuce (Lactuca sativa var. capitata) in Karlsruhe, Germany

**DOI:** 10.1128/MRA.00938-18

**Published:** 2018-08-02

**Authors:** Dominic A. Stoll, Nicolas Danylec, Andreas Dötsch, Biserka Becker, Melanie Huch

**Affiliations:** a Department of Safety and Quality of Fruit and Vegetables, Max Rubner-Institut, Federal Research Institute of Nutrition and Food, Karlsruhe, Germany; b Department of Physiology and Biochemistry of Nutrition, Max Rubner-Institut, Federal Research Institute of Nutrition and Food, Karlsruhe, Germany; Queens College

## Abstract

We report here the genome sequence of Salmonella enterica subsp. enterica serovar Enteritidis MS 501, a potential human pathogen isolated from red lettuce (Lactuca sativa var. capitata) in Karlsruhe, Germany. The assembled genome size was 4,700,322 bp. A total of 4,560 coding genes, 16 rRNAs, 78 tRNAs, and 15 noncoding RNAs were predicted.

## Abstract (precis)

We report here the genome sequence of Salmonella enterica subsp. enterica serovar Enteritidis MS 501, a potential human pathogen isolated from red lettuce (Lactuca sativa var.

## ANNOUNCEMENT

In 2016, 23% of a total of 397 food-related illnesses in Germany were traced back to food contaminated with Salmonella (1). According to the European Food Safety Authority (EFSA), the proportion of confirmed human cases of Salmonella enterica subsp. enterica infection increased from 2012 to 2016 in the European Union, with 59% of 44,462 human salmonellosis cases attributed to S. enterica subsp. enterica serovar Enteritidis (2). Although only 0.21% of 2,429 investigated vegetable samples tested positive for Salmonella (2), it must be remembered that this product group is also intended for raw consumption.

We announce here the draft genome sequence of S. Enteritidis strain MS 501, which was isolated from a sample of red lettuce (Lactuca sativa var. capitata) in 2015. The strain was sent to the National Reference Laboratory of Salmonella at the German Federal Institute for Risk Assessment (BfR) for serotyping. The seroformula is (1),9,12:g,m:-. Genomic DNA was extracted using a blood and tissue kit (Qiagen), and the DNA concentration was determined with a Qubit version 2.0 fluorometer (Thermo Fisher Scientific) according to the manufacturer’s instructions and adjusted to a concentration of 0.2 ng μL^−1^. The sequencing library was built using a Nextera XT DNA library prep kit (Illumina) and a Nextera XT index kit (Illumina), and genome sequencing was performed on an Illumina MiSeq platform using a 600-cycle version 3 kit (read length, 2 × 300 bp). Data processing was done as follows: first, default quality trimming of paired-end reads was performed by Trimmomatic version 0.36 (3). Trimmed reads were assembled using SPAdes version 3.11.1 with default settings in the careful mode (4, 5). The estimated insert size was 263.5 bp. Verification of sufficient trimming was done by mapping the adapter sequences to the assembled contigs using Bowtie2 version 2.3.3.1 (6); to check for contamination, the contigs were aligned to the genome of coliphage PhiX174 (GenBank accession number NC_001422) using a BLASTn search with phi-X174 control sequences (7). All contigs with <500 bp were manually excluded, and renaming of contigs was done by awk (8). Trimmed reads were mapped against the remaining 29 contigs by using Bowtie 2 version 2.3.3.1, which resulted in a coverage of 96.2×. The draft genome sequence was annotated using the automated NCBI Prokaryotic Genome Annotation Pipeline (9) and was scanned for antimicrobial resistance (AMR) genes using ResFinder version 3.0 (10).

The assembly size was 4,700,322 bp, with an *N*_50_ value of 491,907 bp and a GC content of 52.14%. The assembly size is comparable to that of recently announced Salmonella genome sequences (11, 12) and contains 4,560 coding genes, 16 rRNAs, 78 tRNAs, and 15 noncoding RNAs. No AMR gene was identified *in silico*. In addition, no antibiotic susceptibility could be identified phenotypically, as was previously reported (13).

As this potentially pathogenic Salmonella strain was isolated from red lettuce, a fresh produce intended for raw consumption, the surveillance and investigation of these products is necessary to provide knowledge regarding the sources and pathways of such contaminants along the food chain for the development of successful avoidance strategies.

### Data availability.

The draft genome sequence of S. Enteritidis MS 501 has been deposited at DDBJ/ENA/GenBank under BioProject number PRJNA473638.
